# The co‐occurrence of social adversities in early adolescence and their relationship to cognitive outcomes later in development

**DOI:** 10.1002/jcv2.70010

**Published:** 2025-03-28

**Authors:** Man Shiu Kwok, Amber Inman, Kathryn E. Bates, Delia Fuhrmann

**Affiliations:** ^1^ Department of Psychology Institute of Psychiatry, Psychology & Neuroscience King's College London London UK

**Keywords:** adolescents, adversity, ALSPAC, multivariate analysis, social environment

## Abstract

**Background:**

Adverse experiences – such as abuse and neglect – occurring during childhood and adolescence have been found to predict poorer mental and cognitive health. Social adversities, including bullying and social exclusion, are likely to be particularly salient during adolescence. However, our understanding of how social adversities co‐occur in adolescence and how they predict cognitive functioning is limited.

**Methods:**

We used latent profile analysis to investigate adolescents' experiences of social adversities and regressions to identify their relationship to cognition later in development. Data was analysed from UKHLS (*N* = 493, aged 14–16 years, preregistered analysis) and ALSPAC (*N* = 14,856, aged 12–22 years, replication analysis).

**Results:**

Adolescents clustered into four profiles in both cohorts: low adversity, peer difficulties, sibling bullying and poly‐adversity. We found that 17%–22% of participants fell into the poly‐adversity profile and reported experiencing several social adversities. In ALSPAC, but not UKHLS, cognitive functioning differed between social adversity profiles (working memory: *F*(3, 3773) = 8.07, *p* < 0.001; fluid reasoning: *F*(3, 5258) = 3.36, *p* = 0.018; verbal fluency: *F*(3, 5261) = 6.24, *p* < 0.001). After controlling for sex, adolescents in the low adversity profile scored significantly higher on the working memory task than those in the sibling bullying profile and the poly‐adversity profile, but effect sizes were small.

**Conclusion:**

These findings have implications for understanding adolescents' social experiences. To understand individual differences in lifespan outcomes, it is essential to capture a broad spectrum of social interactions, including peer and sibling difficulties, bullying, exclusion, and school issues.

## Graphical abstract

Using latent profile analysis, we investigated the co‐occurrence of adverse experiences in two UK cohorts (UKHLS and ALSPAC, total *N* > 15,000). We identified four profiles in both cohorts: low adversity, peer difficulties, sibling bullying, and poly‐adversity. 17%–22% of participants reported experiencing several social adversities. In ALSPAC, lower working memory and verbal fluency performance were found in adolescents in the poly‐adversity profile compared to the low adversity and sibling bullying profiles.

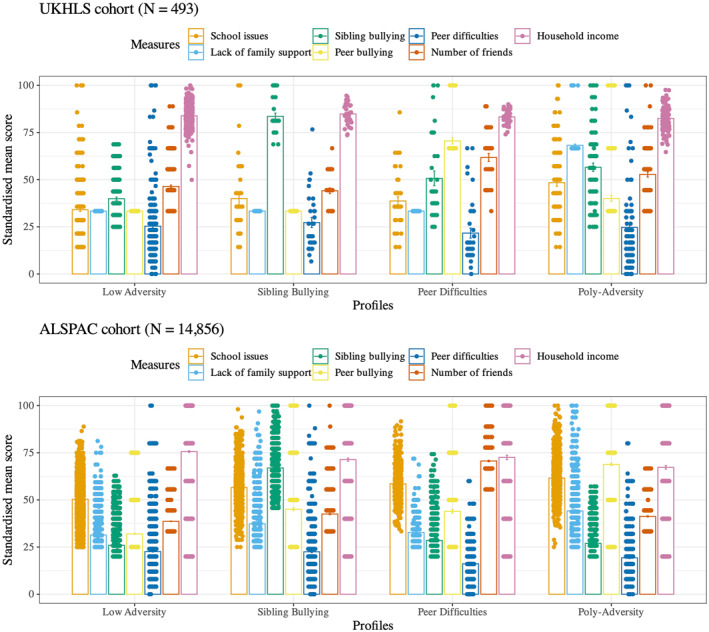

**Abbreviations**
ALSPAC: Avon Longitudinal Study of Parents and Children
UKHLS: UK Household Longitudinal Study: Understanding Society

**Key points**

- The experience of adversity during childhood has previously been found to predict individual differences in mental and cognitive health.
- Less is known about adverse experiences in adolescence (ages 10–24 years) and their relationships to lifespan outcomes.
- We investigated the co‐occurrence of adverse experiences in two UK cohorts (UKHLS and ALSPAC, total *N* > 15,000) using latent profile analysis. We identified four profiles in both cohorts: low adversity, peer difficulties, sibling bullying, and poly‐adversity.
- 17%–22% of participants reported experiencing several social adversities.
- In ALSPAC, but not UKHLS, lower working memory and verbal fluency performance were found in adolescents in the poly‐adversity and sibling bullying profiles compared to the low adversity profile.
- These findings highlight the need to consider social adversities such as peer and sibling bullying and their co‐occurrence in frameworks of adversity in adolescence.
- They also highlight the need for prevention measures tailored to adolescents.

## INTRODUCTION

Adverse experiences are potentially stressful life events, ranging from parental divorce to physical abuse. Childhood adversities have been defined within the Adverse Childhood Experiences (ACEs) framework, which categorises adversities into “child abuse” and “household dysfunction” (Felitti et al., 1998). Around 38.8% of adults have retrospectively reported at least one adverse experience during childhood, and such adversities have been found to account for 29.8% of all DSM–IV disorders across 21 countries (Kessler et al., 2010). National studies indicate that this figure reaches 45% in some countries, including England (Hughes et al., 2020). The experience of adversity during childhood has been found to predict individual differences in brain function and structure (Teicher et al., 2016), as well as poorer physical health (Petruccelli et al., 2019), mental health (Kessler et al., 2010; Pollmann et al., 2022), and cognitive function (Kalia et al., 2021; Kirke‐Smith et al., 2014; Nweze et al., 2023) across the lifespan. The study and identification of adverse childhood experiences have led to effective policy change and improved outcomes for children globally (Edwards et al., 2019).

Less is known about adverse experiences occurring during adolescence (∼10 to 24 years old; Sawyer et al., 2018) and their relationship to cognition. The types of adversity most likely to be salient to adolescents are also likely different from those affecting children. Adolescence is defined as the period of development following the onset of puberty and the development of secondary sex characteristics. Theory and evidence suggest adolescence may be a period of development with heightened sensitivity to social experiences, particularly those related to peer environments (Blakemore & Mills, 2014; Cheng et al., 2024; Fuhrmann et al., 2015; Schaefer et al., 2022). As adolescents mature, peer and intimate relationships become more prominent (Gómez‐López et al., 2019), and adolescents gain more independence in the community through extracurricular activities and seeking employment, for example. Adolescents may, therefore, be at risk of greater exposure to adversities outside of the caregiver environment. Practitioners supporting young people through charities have voiced concern for a range of issues, including financial instability, family conflict, and exposure to online abuse (Inman et al., 2023). A study published in 2022 used network analyses to determine structures of adversities most central to childhood and adolescence. Networks of family and abuse factors were identified in both childhood and adolescence, and a third cluster in adolescence, including educational and social adversities (e.g., being bullied) (Pollmann et al., 2022). Therefore, investigations into social adversities more akin to the lived experience of adolescents are needed.

We here define social adversity as potentially traumatic social experiences. For adolescents, we are particularly interested in social adversities linked to age‐typical environments and relationships. This can include interpersonal adversity, such as bullying, community (e.g., school‐based) and structural level (e.g., socio‐economic status) adversity. This conceptualisation aligns with a recently proposed framework of adolescent adverse experiences emphasising the multi‐level, intersecting systems of adversity (Pollmann et al., 2025). Evidence from childhood indicates that children experiencing one adversity are more likely to experience several others (Bussemakers et al., 2019; McLaughlin et al., 2012). Previous research has examined the relationship between social adversities and adolescent development outcomes, albeit mostly in isolation. For example, a wealth of evidence has highlighted the effect of bullying on adolescent development, with a 2023 systematic review demonstrating that children and adolescents who experienced bullying were 2.77 times more likely to suffer from depression compared to those who were not bullied (Ye et al., 2023). Greater individual‐ and neighbourhood‐level economic hardship has been associated with an increased number of ACEs reported by age 15 (Maguire‐Jack et al., 2021). Recent findings have suggested different levels of social adversity – for example, interpersonal and structural levels – are related. For instance, a study of adolescents aged 13–18 years found socio‐economic status to be negatively associated with peer and family relationships (Li et al., 2020). Taken together, this evidence implies social adversities may be common in adolescents and may interact to shape individual differences, however, this is yet to be formally tested.

ACEs are thought to shape various lifespan outcomes, including cognitive health. Yet, little is known about social adversities and their relationship to cognition in adolescence. ACEs have been found to predict lower cognitive flexibility in college students and adults (Kalia et al., 2021) and lower executive functioning in adolescence (Kirke‐Smith et al., 2014). A study published in 2023 examined profiles of adversity in childhood (from 8 months to 8 years of age) and tested how they predicted cognitive outcomes at 24 years in the ALSPAC sample. They found five classes of adversity: low adversity, dysfunctional family, parental deprivation, family poverty and global adversity, with nearly 30% of the sample classified into adversity profiles. Children classified into adversity profiles (e.g., family poverty and global adversity profiles) showed lower performance on cognitive tasks than those in the low adversity profile (Nweze et al., 2023). Multivariate approaches to understanding the relationship between adversities and adolescent cognition have also emerged in recent preprints investigating, for example, profiles of socio‐economic disadvantages (Shariq et al., 2024, June 10). This research highlights that adopting a multivariate approach can help identify subgroups of individuals who might be at risk of poorer cognitive functioning in adulthood.

### The current study

This review of the current literature suggests that research is needed to capture the co‐occurrence of adversities and their relationship to cognition. There is a particular research gap in understanding adverse experiences and their co‐occurrence in adolescence, a formative period of development. To complement the evidence for childhood, we here use a multivariate approach characterising profiles of adolescent social adversities. This allows us to identify potential subgroups of adolescents who are more vulnerable to different or multiple social adversities. Our first aim is, therefore, to identify profiles of young people based on their experience of social adversities and how the experiences of different social adversities co‐occur. Our second aim is to identify relationships between profiles of adverse experiences and cognition. Here, we use data from two UK cohorts: UKHLS (preregistered analysis) and ALPSAC (replication analysis). We used a person‐centred, multivariate latent profile analysis approach to examine the co‐occurrence of social adversities during early adolescence. We investigated how social adversity profiles predict cognitive outcomes in later adolescence. Latent profile analysis is a data‐driven approach that clusters participants into profiles using probabilistic assignment (Spurk et al., 2020). It allowed us to identify subgroups of adolescents based on their experience of multiple social adversities. Our approach captures constructs at multiple levels of adversity: interpersonal (sibling bullying, peer bullying, number of friends, lack of family support), community (school issues), and structural (household income) levels of the environment. This is in line with Bronfenbrenner's ecological systems theory that multiple levels of systems in the environment interact with one another to shape development (Bronfenbrenner, 1979). Recent research has demonstrated the intersecting levels of adversity in adolescent development and mental health (Pollmann et al., 2022, 2025). We hypothesised that (a) There will be distinct profiles of social adversity in early adolescence and (b) These profiles will predict cognitive abilities in the same individuals later in adolescence.

## METHODS

### Cohorts

In the current study, we conducted preregistered analyses of data collected from the UKHLS (University of Essex, Institute for Social and Economic Research, 2022) and then replicated the study in ALSPAC (Boyd et al., 2013; Fraser et al., 2013; Northstone et al., 2019).

For UKHLS, study data from waves 1–3 were mainly collected through face‐to‐face interviews, and a small proportion of data was collected via phone interviews. During interviews, participants responded to questions asked by the interviewers and completed a questionnaire. Further information on the cohort and data access information can be found on the Understanding Society website: https://www.understandingsociety.ac.uk.

For ALSPAC, study data were collected and managed using REDCap (Research Electronic Data Capture) electronic data capture tools (Harris et al., 2009) hosted at the University of Bristol. REDCap is a secure, web‐based software platform designed to support data capture for research studies. The study website contains information on data access and details of all the data that is available through a fully searchable data dictionary and variable search tool: http://www.bristol.ac.uk/alspac/researchers/our‐data/.

#### UKHLS

For the UKHLS dataset, we selected participants who were 14 years old at wave 1 (January 2009 – March 2011) and who had also completed wave 3 (January 2011– July 2013) at 16 years old. This resulted in 493 (Female = 255, Male = 238) participants.

#### ALSPAC

Pregnant women resident in Avon, UK, with expected delivery dates between 1st April 1991 and 31st December 1992, were invited to participate in the ALSPAC study. The total sample size for analyses using any data collected after age seven is 15,447 pregnancies, resulting in 15,658 foetuses. For our purposes, we selected participants who joined data collection before they were 18 (phase VI) and contained information for at least one of our variables of interest. This resulted in 14,856 (Female = 7,217, Male = 7,618, Missing = 21) participants. Due to limited relevant variables in the ALSPAC data at age 14, we used data collected retrospectively at age 12–22 to match adversity and cognitive measures to our first study as closely as possible.

### Measures

#### Social adversities

We selected seven variables to capture interpersonal, community and structural levels of adversity exposure from the UKHLS dataset: peer bullying, peer relationship problems, number of close friends, school issues, lack of family support, sibling bullying, and household income. Multiple items were used to measure peer relationship problems, school issues, and sibling bullying. We preregistered the computation of sum scores for variables with multiple items; however, we decided to compute mean scores instead in case some items had missing data. Social adversities are described here, and the variable names are recorded in Table S1 of the Supporting Information S1 to aid replication. Peer bullying was measured by how frequently the participant was picked on or bullied by their peer. The three items for peer relationship problems measure the extent to which the participant felt they are always alone, liked by others of the same age, and get along with adults better than those of their age. The number of close friends was the total number of close friends participants reported having (up to 100). Items that captured school issues focussed on how the participants felt about schoolwork and their school. The lack of family support was measured by the support the participant reported receiving from their family. The four items for sibling bullying captured instances of both verbal and physical bullying. Participants' familial socioeconomic status was measured by monthly household income, which was log‐transformed. The transformation was not preregistered but was needed to achieve normalisation.

For the replication in ALSPAC, we selected items as close in age and content to the seven social adversity variables in UKHLS as possible, given the available data (see Supporting Information S1: Table S1). In ALSPAC, peer bullying was measured at age 11 years, the number of close friends was measured at age 13.5 years, and the lack of family support was measured retrospectively at age 22. The other four social adversity variables and lack of family support are measured with multiple items, for which we derived a mean score across items. Peer bullying was measured by the frequency of the participant feeling upset due to name‐calling, exclusion or bullying from peers. Peer relationship problems were captured by six items regarding how the participants feel and get along with people in school. Thirteen items were administered to measure participants' thoughts and feelings towards school for school issues. The lack of family support was measured by eight items capturing verbal and physical conflict in the household between the ages of 11 and 17. For sibling bullying, we captured physical and verbal bullying with seven items at age 12. Finally, the Index of Material Deprivation Income Score was used to measure the participants' socioeconomic status.

#### Cognitive functioning measures

Cognitive functioning measures included working memory, fluid reasoning, and verbal fluency. We selected measures to capture verbal and fluid domains, which is in line with previous research investigating the relationship between adversity and cognition (Kirke‐Smith et al., 2014; Nweze et al., 2023). Working memory was selected in light of previous research suggesting social exclusion in adolescence was associated with poorer working memory (Fuhrmann et al., 2019). UKHLS measured the cognitive functioning of participants at wave 3 when participants were 16 years old. Meanwhile, ALSPAC measured different cognitive abilities over multiple time points. Therefore, we used the data collected at ages 15.5 (fluid reasoning and verbal fluency) and 17.5 (working memory) in ALSPAC to match the cognitive measures we investigated in UKHLS. Variable names are provided in Supporting Information S1: Table S2 to aid replication.

##### Working memory

In UKHLS, the Serial 7 Subtraction test was used to measure the visuospatial working memory (Fisher et al., 2014). Participants had to subtract 7 from a series of numbers starting from 100, without any material or aids. For every response that was 7 less than the previous answer, a score of 1 was recorded. This was repeated until five trials were completed, and the maximum score for the test was 5.

In ALSPAC, participants completed the N‐Back task to assess their working memory at 17.5 years old. During the test, a series of stimuli were presented to the participant. Participants had to monitor the stimuli presented to them and respond whenever the stimulus presented was identical to the stimulus presented in N trials previously. All participants completed the N‐back task with N specified as 1, 2 or 3. Participants completed 48 trials for each condition. Results from the 2‐back and 3‐back conditions indicate the ability to hold previous information in mind.

##### Fluid reasoning

UKHLS participants were assessed on their fluid reasoning with the number series task (Fisher et al., 2014). Participants were randomly assigned to one out of two sets of the number series task. Participants had to answer which number should be in the blank among the given sequence of numbers. A score of one was awarded for each correct answer, and the total number of trials was 15.

In ALSPAC, fluid reasoning was measured using the matrix reasoning test from the Wechsler Abbreviated Scale of Intelligence (WASI) (Wechsler, 1999) and was administered at 15.5 years old. In this task, a series of stimuli with a blank stimulus was presented to the participants during the test. Participants then had to select a stimulus out of five options that would fit in the given sequence. A score of one was awarded to each correct answer; there were 33 questions in total.

##### Verbal fluency

UKHLS and ALSPAC measured the verbal ability of participants with verbal fluency and verbal intelligence, respectively. In UKHLS, the verbal fluency test was from the English Longitudinal Study on Ageing (Llewellyn & Matthew, 2009) and was used to assess semantic or category fluency. Participants were asked to name as many animals as they could within 60 s, and a score of one was awarded for each correct answer.

ALSPAC used the WASI test (Wechsler, 1999) to measure the verbal intelligence of participants at 15.5 years old. The interviewer asked the participants to define 28 words if they were under 16 and 32 words if they were over 16. Furthermore, participants were asked to identify what the given five images showed.

### Data analysis

The data analysis procedure was preregistered for the UKHLS cohort. The preregistration can be accessed here: https://osf.io/52chx/. We then conducted a conceptual replication of the study in ALSPAC, which was not preregistered. All statistical analyses were performed using Rstudio (R version 4.1.1) (R Core Team, 2021). The R script is available on the OSF: https://osf.io/52chx/. See Supporting Information S1 for data wrangling steps and Supporting Information S1: Tables S3 and S4 for a breakdown of missingness. We used *tidyLPA* (Rosenberg et al., 2018) to estimate the number of adversity profiles using latent profile analysis. The number of profiles that best fit the data was determined using the following criteria (Tein et al., 2013, p. 20): (a) low Sample Adjusted Bayesian Information Criteria (SABIC) indicating better model fit, (b) entropy closer to 1 indicating a higher probability of distinct groups, and (c) a significant *p*‐value (*p* < 0.05) for the Bootstrapped Likelihood Ratio Test (BLRT) indicating that *k* profiles fit the data better than *k* − 1 profiles. In non‐preregistered analyses, we conducted pairwise comparisons between all profiles on the estimated marginal means of each measure with the *emmeans* package (Lenth et al., 2023). We applied Bonferroni corrections to multiple comparisons, correcting for 42 comparisons. The effect size was obtained by computing each comparison's Cohen's *d* statistic.

Using the car package (Fox & Weisberg, 2019), we then conducted regression analyses with cognitive measures as the dependent variable and profiles and sex as the independent variables as pre‐registered. Regression analyses without sex as a covariate are reported as a sensitivity analysis in the Supporting Information S1. To run the regressions with categorical variables as predictors, we used Helmert‐coding to code for contrasts between levels. Pair‐wise comparisons were corrected for 18 comparisons using Bonferroni correction. Effect sizes were obtained by computing each comparison's Cohen's *d* statistic.

## RESULTS

**FIGURE 1:**
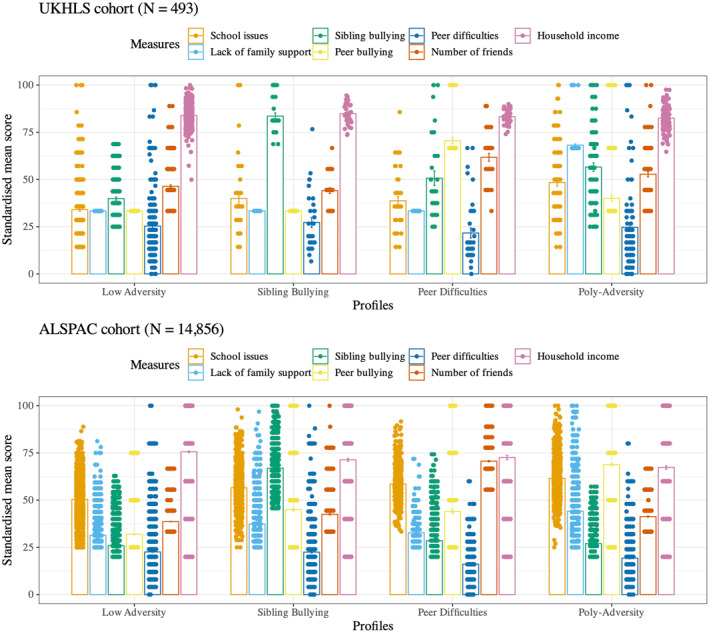
Mean, standard error (SE) and individual data points of social adversities for each profile for the UKLHS cohort (top) and ALSPAC cohort (bottom).

For both cohorts, four‐profile solutions best fit the data (see Supporting Information S1: Tables S5 and S6). In UKHLS, the four profiles were classified as low adversity (*N* = 306, 62% of the sample), sibling bullying (*N* = 35, 7% of the sample), peer difficulties (*N* = 43, 9% of the sample) and poly‐adversity (*N* = 109, 22% of the sample) (see Figure 1). To better understand the characteristics of each profile, we conducted a non‐preregistered exploratory analysis comparing the social adversity scores between profiles. The full results are reported in Supporting Information S1: Table S7. The low adversity profile was characterised by low scores across adversity measures. The poly‐adversity profile was characterised by high scores across all adversities compared to the low adversity profile (*ps* < 0.001, *α*_*Bonf.*_ = 0.001) except for number of close friends (*p* = 0.755, *α*_*Bonf.*_ = 0.001) and monthly income (*p* = 0.044, *α*_*Bonf.*_ = 0.001). The sibling bullying profile was characterized by higher sibling bullying scores compared to all other profiles (*ps* < 0.001, *α*_*Bonf.*_ = 0.001). The peer bullying difficulties profile was characterized by higher peer bullying and peer relationship problem scores compared to all other profiles (*ps* < 0.001, *α*_*Bonf.*_ = 0.001).

In ALSPAC, the four profiles were consistent with the UKHLS cohort: low adversity (*N* = 8,592, 58% of the sample), sibling bullying (*N* = 2,542, 17% of the sample), peer difficulties (*N* = 1,265, 9% of the sample) and poly‐adversity (*N* = 2,457, 17% of the sample) (see Figure 1). Like the profiles in UKHLS, the low adversity profile was characterised by low scores across adversity measures. The sibling bullying profile had significantly higher mean sibling bullying scores than all other profiles (*ps* < 0.001, *α*_*Bonf.*_ = 0.001). The peer difficulties profile was characterised by significantly higher mean scores in peer‐related adversities, compared to all other profiles (*p*s < 0.001, *α*_*Bonf.*_ = 0.001), other than for the comparison between the sibling bullying and peer difficulties profile for peer bullying (*p* = 0.002, *α*_*Bonf.*_ = 0.001). The poly‐adversity profile had significantly higher mean scores (*p*s < 0.001, *α*_*Bonf.*_ = 0.001) than the low adversity profile across all adversity measures other than lack of family support (*p* = 0.028, *α*_*Bonf.*_ = 0.001) and monthly income (*p* = 0.013, *α*_*Bonf.*_ = 0.001). The full results are reported in Supporting Information S1: Table S8.

We next ran regression models controlling for sex to examine whether there were differences in cognitive functioning later in adolescence between different profiles of social adversity. Results from the regression models indicated that social adversity profiles at age 14 did not significantly predict cognitive functioning at age 16 in the UKHLS dataset (working memory: *F*(3, 411) = 0.93, *p* = 0.427; fluid reasoning (*F*(3, 408) = 0.85, *p* = 0.466) and verbal fluency (*F*(3, 416) = 0.57, *p* = 0.634). In ALSPAC, the main effect of social adversity profile on each cognitive functioning was significant: working memory (*F*(3, 3773) = 8.07, *p* < 0.001), fluid reasoning (*F*(3, 5258) = 3.36, *p* = 0.018) and verbal fluency (*F*(3, 5261) = 6.24, *p* < 0.001). The full regression models are reported in Supporting Information S1: Table S9. Pair‐wise comparisons are reported in Table 1. After controlling for sex, adolescents in the low adversity profile scored significantly higher on the working memory task than those in the sibling bullying profile and the poly‐adversity profile. Participants in the peer difficulties profile scored significantly higher than those in the poly‐adversity profile and sibling bullying profile. For fluid reasoning, there were no significant differences in scores after correcting for multiple comparison. For verbal fluency, participants in the low adversity profile scored significantly higher than those in the sibling bullying profile and those in the poly‐adversity profile. Effect sizes were very small or small throughout.

**TABLE 1:** Estimated marginal means pairwise comparisons of cognitive outcomes between social adversity profiles in ALSPAC.

		Contrasts					
Cognitive measure		1 versus 2	1 versus 3	1 versus 4	2 versus 3	2 versus 4	3 versus 4
Working memory	Est.	2.88	−1.34	3.54	−4.22	0.66	4.88
	SE	0.90	1.23	0.95	1.41	1.17	1.44
	df	3773	3773	3773	3773	3773	3773
	*t*‐ratio	3.21	−1.10	3.74	−3.01	0.57	3.40
	*p*	**0.001**	0.273	**<0.001**	**0.0027**	0.572	**0.001**
	*d*	0.15	−0.07	0.18	−0.21	0.03	0.25
Fluid reasoning	Est.	0.20	0.97	1.45	0.77	1.26	0.49
	SE	0.46	0.61	0.49	0.70	0.60	0.73
	df	5258	5258	5258	5258	5258	5258
	*t*‐ratio	0.43	1.58	2.94	1.10	2.10	0.67
	*p*	0.669	0.114	0.003	0.271	0.036	0.504
	*d*	0.01	0.05	0.07	0.04	0.06	0.02
Verbal fluency	Est.	1.58	1.36	1.90	−0.22	0.32	0.54
	SE	0.52	0.69	0.55	0.79	0.67	0.81
	df	5261	5261	5261	5261	5261	5261
	*t*‐ratio	3.06	1.98	3.43	−0.28	0.48	0.67
	*p*	**0.002**	0.048	**0.001**	0.780	0.631	0.505
	*d*	0.08	0.07	0.10	−0.01	0.02	0.03

*Note*: The model included sex as a covariate. 1 = low adversity profile, 2 = sibling bullying profile, 3 = peer difficulties profile, 4 = poly‐adversity profile. *p‐*values in bold indicate significance after Bonferroni correction (adjusted alpha = 0.0028).

We also fit the models without sex as a covariate as a non‐preregistered sensitivity analysis (Supporting Information S1 and Supporting Information S1: Table S9, pairwise comparisons reported in Supporting Information S1: Table S10). These models showed the same pattern of results. The social adversity profile was not a significant predictor of cognitive outcomes in UKHLS but was a significant predictor of cognitive outcomes in ALSPAC. See Supporting Information S1: Tables S11 and S12 for descriptives and Supporting Information S1 for details of assumptions checked for the regression analysis. Spearman's correlation tables for adversity variables in each cohort are presented in Supporting Information S1: Tables S13 and S14.

## DISCUSSION

In this study, we used a person‐centred, multivariate approach to investigate the co‐occurrence of social adversities in adolescence in two UK cohorts. We tested whether distinct profiles of social adversities predicted cognitive outcomes in later adolescence. Results from our latent profile analysis revealed individual differences in experiences of social adversities: There were four different profiles (low adversity, sibling adversity, peer difficulties, and poly‐adversity) in both the UKHLS and ALSPAC cohorts. Notably, 17%–22% of participants reported experiencing several social adversities (poly‐adversity profile), highlighting the utility of multivariate, person‐centred approaches to understanding young people's experience of social adversity. Social adversity profiles did not predict later cognitive abilities in the UKHLS cohort. In the ALSPAC cohort, social adversity profiles predicted cognitive functioning. Higher working memory and verbal fluency were found in the low adversity profile compared to the poly‐adversity and sibling bullying profiles. Higher working memory was also found in the peer difficulties profile compared to the sibling bullying and poly‐adversity profiles. However, the effect sizes were very small to small. These findings highlight the high occurrence of multiple and different social adversities in some adolescents. They also indicate that some adolescents who have experienced several social adversities in early adolescence may be at a slightly higher risk of poorer cognitive function in later adolescence. This highlights the need for directing support towards early adolescence to reduce the prevalence of social adversities and mitigate the risk to later cognitive function.

The converging results of finding four distinct profiles in two independent datasets (UKHLS and ALSPAC) suggest specific patterns of social adversities and the high co‐occurrence of social adversities during adolescence for some young people. Notably, some correlations between social adversities were small at the cohort level. This contrasts with the latent profile approach, which allowed us to identify distinct profiles based on patterns of co‐occurrence and, therefore, capture the meaningful heterogeneity in the way that adolescents experience social adversities. Overall, this pattern of results highlights the complementary nature of variable‐centred and person‐centred approaches. Using latent profile analysis, we identified four distinct profiles in both datasets: low adversity, sibling bullying, peer difficulties and poly‐adversities. Previous studies examining the profiles of ACEs in children or other developmental samples (e.g., birth to 19 years) have found that the majority of participants cluster into a low adversity profile, and around 10% of participants cluster into poly‐adversity profiles (Brieant et al., 2022; Lacey et al., 2022; Nweze et al., 2023). We saw a similar pattern of results in how adolescents experience social adversities. However, the proportion of adolescents reporting the experience of several social adversities is much higher at 17%–22% (poly adversity profile). This indicates that social adversities may be more prominent in adolescence and highlights the importance of understanding a broad set of adversities for adolescence (Inman et al., 2023, Pollmann et al., 2025).

There was a high prevalence of sibling bullying reported by adolescents in both cohorts. In our study, 7% of adolescents were classified into the sibling bullying profile in the UKHLS sample and 17% in the ALSPAC sample. Previous studies have found that childhood sibling bullying predicts important outcomes such as depression and self‐harm (Bowes et al., 2014). Despite this evidence, sibling bullying often receives less consideration than peer bullying and can be brushed off as “normal” (Khan & Rogers, 2015). A study published in 2022 investigated the longitudinal and concurrent effects of sibling bullying on mental health in adolescents aged 11, 14, and 17 years. They found sibling bullying at age 11 predicted poorer mental health later in adolescence. Notably, those who experienced repeated victimisation at 11 and 14 years reported higher internalizing and externalising issues, higher rates of self‐harming, and lower well‐being and self‐esteem at 17 years than those experiencing sibling bullying at 11 or 14 years (Toseeb & Wolke, 2022). Together, these studies emphasise how salient sibling bullying can be to adolescents.

We found that social adversity profiles predicted cognitive outcomes in the ALSPAC dataset but not in the UKHLS dataset. The results suggest individual differences in the relationship between social adversities and cognition for ALSPAC with lower working memory and verbal fluency in adolescents in the poly‐adversity profile compared to low adversity. While effect sizes were small, this is broadly consistent with evidence from studies investigating caregiver adversities in childhood. For example, adversity during childhood was found to predict lower working memory in adolescence (Nweze et al., 2023). We did not find evidence for this relationship in the UKHLS cohort; however, this could be due to smaller sample sizes or other differences between cohorts, including differences in measurement and age ranges studied. Further research is needed to better understand how individual differences in social adversities might shape cognitive function across three or more time points (Parsons & McCormick, 2022).

There are several strengths to this study: We conducted this study using publicly available data from the UKHLS cohort study with a preregistered analysis plan and replicated the study in a second large‐scale cohort, ALSPAC. We have provided detailed information in the Supporting Information S1 on the variables computed with the item names from each dataset to aid replication. We leveraged a multivariate approach to understand the prevalence and co‐occurrences of social adversities at different system levels (interpersonal, community, and structural), which is akin to the multifaceted social experiences of adolescents. There are some limitations to consider, too: There are differences between cohorts in terms of sample sizes and age ranges studied. Adversity and cognition measures were different between the two studies. Therefore, while we have provided a conceptual replication, not an exact replication. Collaboration between cohort studies in variable selection should be considered in future to facilitate replication research. It is also important to note that caution is required when interpreting developmental/longitudinal effects with only two time points of repeated measures data (Parsons & McCormick, 2022). The research reported here is also observational and does not allow for causal inference. Finally, we have had to make some deviations from our preregistered plans. For instance, we computed mean scores rather than sum scores due to missing data. We have made these deviations transparent in the methods section.

## CONCLUSION

The current study provides evidence for individual differences in the experience of social adversities during adolescence. We investigated how profiles of social adversity in early adolescence predict cognitive outcomes in later adolescence. Evidence for individual differences in experiences of adversity was robust across cohorts and analyses. Around 17%–22% of adolescents across two large‐scale cohort studies reported experiencing several social adversities, and the experience varies across individuals. Some experience peer‐related adversities, such as peer bullying, and others family related adversities, such as sibling bullying. This has methodological implications for measuring adolescent adversity in future studies: we need to capture adversity at the individual, community, and structural levels rather than only focussing on peer relationships. The evidence presented here also indicates that for some individuals, the experience of social adversities may put them at risk for later poorer cognitive functioning; future research is needed to disentangle longitudinal effects. Overall, these findings highlight that future research into adversity prevention should consider a range of peer and sibling difficulties, including bullying, exclusion, and school issues.

## AUTHOR CONTRIBUTIONS

**Man Shiu Kwok**: Data curation; formal analysis; methodology; visualization; writing—original draft. **Amber Inman**: Data curation; formal analysis; methodology; visualization; writing—original draft. **Kathryn E. Bates**: Conceptualization; data curation; formal analysis; methodology; project administration; supervision; visualization; writing—review and editing. **Delia Fuhrmann**: Conceptualization; funding acquisition; supervision; writing—review and editing.

## CONFLICT OF INTEREST STATEMENT

The authors declare no conflicts of interest.

## ETHICAL CONSIDERATIONS

*UKHLS:* The University of Essex Ethics Committee has approved all data collection on Understanding Society's main study, COVID‐19 surveys and innovation panel waves, including asking for consent for all data linkages except to health records. Requesting consent for health record linkage was approved at Wave 1 by the National Research Ethics Service (NRES) Oxfordshire REC A (08/H0604/124), at BHPS Wave 18 by the NRES Royal Free Hospital and Medical School (08/H0720/60) and at Wave 4 by NRES Southampton REC A (11/SC/0274). Approval for asking consent for health record linkage and for the collection of blood and subsequent serology testing in the March 2021 wave of the COVID‐19 study was obtained from the London‐City and East Research Ethics Committee (21/HRA/0644). Approval for the collection of biosocial data by trained nurses in Waves 2 and 3 of the main survey was obtained from the National Research Ethics Service (Understanding Society ‐ UK Household Longitudinal Study: A Biosocial Component, Oxfordshire A REC, Reference: 10/H0604/2). The biosocial data collection at IP12 ‘Understanding Society Health Innovation Panel: Biomeasure and health data collection from the Innovation Panel of the UK Household Longitudinal Study’ was approved by East of England ‐ Essex Research Ethics Committee, Ref 19/EE/0146. *ALSPAC:* Ethical approval for the study was obtained from the ALSPAC Ethics and Law Committee and the Local Research Ethics Committees. Informed consent for the use of data collected via questionnaires and clinics was obtained from participants following the recommendations of the ALSPAC Ethics and Law Committee at the time.

## Supporting information

Supplementary Material

## Data Availability

The data supporting this study's findings are available from the data owners (ALSPAC and UKHLS) via data access requests.
