# Supplementary material for: The co‐occurrence of social adversities in early adolescence and their relationship to cognitive outcomes later in development
Source: JCPP Adv. 2025 Mar 28;5(4):e70010. doi: 10.1002/jcv2.70010 (PMC12698274; doi:10.1002/jcv2.70010)
Supplement: Supplementary file 1 — Supplementary Material [file JCV2-5-e70010-s001.docx]

**Supporting Information**

**Supplementary Methods**

***Data wrangling***

Plots were generated using the package *ggplot2* (Wickham, 2009) and ggpubr (Kassambara, 2023). Before conducting the analysis, we selected the participants from the full datasets using the packages *tidyverse* and *dplyr* (Wickham et al., 2019, 2023). We transformed all measures to standardised measures of 0-100, where each score represents the percentage of the maximum score observed on a variable. This step was not pre-registered but was required to facilitate interpretation and model convergence in latent profile analysis (LPA). As preregistered, outliers were identified as univariate z scores +/- 5 and replaced with NA (UKHLS: 3 participant responses for the number of close friends and household income, ALSPAC: 25 participant responses on the number of close friends, 10 and 28 participant scores on peer relationship problems and lack of family support, respectively). As pre-registered, all missing data were handled through a single imputation in LPA; this choice was made because multiple imputations may distort profile classification in LPA (Sterba, 2016).

**Supplementary Results**

***Determining the number of profiles***

To examine how many profiles of social adversities best fit the data, we estimated models with one to six profiles (see Table S4). In the UKHLS dataset, there was less unexplained variance in the model as the number of profiles increased from one to four, as indicated by the SABIC. The BLRT *p*-value also suggested that the four-profile solution fit the data better than the 3-profile solution (*p* = .01). The four-profile solution showed a high probability of accurate group separation indicated by entropy (.91). Therefore, the four-profile model was chosen as the final model. In the ALSPAC dataset, the SABIC indicated that there was less unexplained variance in the model as the number of profiles increased from one to six and the BLRT *p*-value remained significant (*p* = .009). In contrast to the entropies obtained from the UKHLS LPA models, the entropies of the LPA models were relatively low in the ALSPAC cohort. This is likely due to the larger sample size in the ALSPAC cohort (Wang et al., 2017). While the three-profile solution showed a higher probability of accurate group separation than the four-profile solution (higher entropy), the number of participants in one of the profiles of the three-profile solution equated to only 2% of the sample (5% is recommended as a minimum: Aflaki et al., 2022). Although the five-profile and four-profile solutions showed similar fit statistics (see Table S4), the five-profile solution contains a profile with only 4% of the population, while the four-profile solution has a comparatively more balanced profile prevalence (see Table S5). Therefore, the four-profile model was selected as the final model.

***Regression assumptions check.***

The assumption of linearity was not violated for all regression analyses by design as we used Helmert coding for the four levels of the social adversity profile. In UKHLS, the Shapiro-Wilk normality test of residuals was not significant for verbal fluency (*W* = .99, *p* = .179), but was significant for working memory (*W* = .65, *p* < .001) and fluid reasoning (*W* = .98, *p* = < .001). As the sample size in ALSPAC exceeded 5000, we used the one-sample Kolmogorov-Smirnov test. Results showed that the assumption of normality of residuals was violated for all linear regression models in ALSPAC: working memory (*D* = .48*, p* < .001), fluid reasoning (*D* = .49, *p* < .001), verbal fluency (*D* = .46, *p* < .001). However, regression models are typically robust to violations of normality (Schmidt & Finan, 2018).

***Regression models without sex as a covariate***

As a sensitivity check, we fit the regression models without sex as a covariate. As in the main analysis, there was no main effect of social adversity profile on any of the cognitive functioning measures in the UKHLS dataset (working memory: *F*(3, 412) = .936, *p* = .423); fluid reasoning: *F*(3, 409) = 1.02, *p* = .382; verbal fluency: *F*(3, 417) = .493, *p* = .688). In ALSPAC, the main effect of social adversity profile on each cognitive domain was significant: working memory (*F*(3, 3777) = 8.47, *p* < .001), fluid reasoning (*F*(3, 5268) = 3.48, *p* = .015) and verbal fluency (*F*(3, 5271) = 6.44, *p* < .001). The full regression model is reported in the Supplementary Table S9. All pairwise comparisons for ALSPAC are reported in Table S10. Pairwise comparisons showed a very similar pattern of results as the main analysis. The only difference was that for fluid reasoning, the low adversity profile scored significantly higher than the poly-adversity profile here. As in the main analysis, effect sizes were very small or small throughout.

**Supplementary Tables**

*Table S1.* Social adversity measures in UKHLS and ALSPAC

| **Adversity Measure** | **Item(s)** | |
| --- | --- | --- |
|  | *UKHLS* | *ALSPAC* |
| Peer Bullying | a_ypsdqs: Other children or young people pick on or bully | *ccxa210: Frequency during the last school year YP has been upset by name calling/exclusion from groups or bullying* |
| Peer Relationship Problems | a_ypsdqf: Usually on own. Generally plays alone or keeps to themselves ^a^  *a_ypsdqn: Generally liked by others own age ^a^*  a_ypsdqw: Gets on better with adults ^a^ | fg4128: Teenager believes friends understand them  fg4129: Teenager talks about problems with friends  fg4130: Teenager is happy with their friends |
| Number of Friendships | a_ypnpal: Number of close friends | fg4121: Number of close friends teenager has |
| School Issues | a_yphsc: How do you feel about the school you go to  a_yphsw: How do you feel about your schoolwork ^c^ | ccr100: Respondent's school is a place where they really like to go each day  ccr102: Respondent's school is a place where other pupils accept them  ccr103: Respondent's school is a place where they like to be  ccr104: Respondent's school is a place where they like to do extra work  ccr105: Respondent's school is a place where they feel happy  ccr107: Respondent's school is a place where they feel proud to be a pupil  *ccr108: Respondent's school is a place where they feel worried*  ccr110: Respondent's school is a place where they have a lot of fun  ccr111: Respondent's school is a place where they enjoy what they do in class  ccr112: Respondent's school is a place where they can learn what they need to know  ccr113: Respondent's school is a place where they get excited about the work they do  *ccr114: Respondent's school is a place where they get upset*  *ccr119: Respondent's school is a place where they feel restless* |
| Lack of Family Support | a_ypfamsup: Feel supported by family – that is those that live with you | YPB8050: Frequency adult in family shouted at respondent between ages of 11 and 17  YPB8051: Frequency adult in family said hurtful or insulting things to respondent between ages of 11 and 17  YPB8052: Frequency adult in family pushed, grabbed or shoved respondent between ages of 11 and 17  YPB8053: Frequency adult in family smacked respondent for discipline between ages of 11 and 17  YPB8054: Frequency adult in family punished respondent in a way that seemed cruel between ages of 11 and 17  YPB8055: Frequency adult in family threatened to kick, punch, hit respondent with something that could hurt respondent or physically  YPB8056: Frequency adult in family actually kicked, punched, hit respondent with something that could hurt respondent or physically  YPB8057: Frequency adult in family hit respondent so hard it left bruises or marks between ages of 11 and 17 |
| Sibling Bullying | a_ypsibhit: Whether brothers or sisters hit kick or push  a_ypsibsteal: Whether brothers or sisters take belongings  a_ypsibtease: Whether brothers or sisters make fun of you  a_ypsibverab: Whether brothers or sisters call you nasty names | *ccl201: Frequency child is bullied by sibling*  *ccl210: Frequency child is hit, kicked, pushed or shoved by sibling*  *ccl211: Frequency child has things damaged or taken from them by sibling*  *ccl212: Frequency child is called names by sibling*  *ccl213: Frequency child is made fun of by sibling*  *ccl214: Frequency child is ignored or left out of siblings games or social groups*  *ccl215: Frequency sibling has told lies or spread rumours about child* |
| Familial Socioeconomic Status (SES) | a_fihhmnnet1_dv: Net household monthly income – no deductions ^c^ | *ccrimd2015_incomeq5: IMD Income Score 2015* |

*Note. Items in italics are reverse coded in the analysis to ensure items are scored in one direction.*

^a^ Although we pre-registered to use a_ypsdqpp: SDQ Subscale: Peer Relationship Problems as the measurement of peer relationship problem, we found that it includes peer bullying and number of close friends in the scale. Therefore, we have used items that do not overlap between variables.

^b^ School issues is used here and in the main manuscript, instead of educational issues used in the preregistration.

^c^ In the pre-registration, we mistyped Yphsw as Yphsw and a_fihhmnnet1_dv as W_fihhmnnet_dv

*Table S2.* Cognitive measures in UKHLS and ALSPAC

| **Outcome** | **Task** | **Item(s)** |
| --- | --- | --- |
| *UKHLS* | | |
| Working Memory | Subtract 7 | c_cgs7cs_dv: Cognitive ability: Subtract 7: Number of correct answers ^b^ |
| Fluid Reasoning ^a^ | Number Series | c_cgns1sc6_dv: Cognitive ability: Number series: Set 1 A+B total score ^b^  c_cgns2sc6_dv: Cognitive ability: Number series: Set 1 A+B total score ^c^ |
| Verbal Fluency | Animal Task | c_cgvfc_dv: Cognitive ability: Verbal fluency: Count of correct answers |
| *ALSPAC* | | |
| Working Memory | N Back Task | FJNB100: Mean accuracy to identify targets for the two back procedure  FJNB300: Mean accuracy to identify targets for the three back procedure |
| Fluid Reasoning ^a^ | Matrix Reasoning | fh6275: Raw score for Matrix Reasoning subtest |
| Verbal Fluency | Vocabulary Test | fh6272: Raw score for Vocabulary subtest |

^a^ Fluid reasoning is used here, instead of fluid reasoning used in the preregistration.

^b^ The items c_cgs7cs_dv and c_cgns1sc6_dv were misspelled in the preregistration (incorrect: c_cgs7ca_dv and c_cns1sc6_dv).

^c^ There were two sets of number series task and participants were split between the two sets randomly; both sets are included here but set 2 was missed from the preregistration in error.

*Table S3.* Frequency of missing data in the UKHLS cohort

| **Measures** | **Missing frequency** | **Percentage missing (%)** |
| --- | --- | --- |
| Peer Bullying | 3 | 0.61 |
| Peer Relationship Problem | 2 | 0.41 |
| Number of Close Friends | 28 | 5.68 |
| School Issues ^a^ | 0 | 0 |
| Lack of Family Support | 2 | 0.41 |
| Sibling Bullying ^b^ | 73 | 14.81 |
| Monthly Household Income | 3 | 0.61 |
| Working Memory | 77 | 15.62 |
| Fluid Reasoning ^c^ | 80 | 16.23 |
| Verbal Fluency | 72 | 14.60 |

^a^ School issues is used here and in the main manuscript, instead of educational issues in the preregistration.

^b^ There is a high percentage of missing data for sibling bullying items since 72 participants reported that they do not have a sibling.

^c^ Fluid reasoning is used here and in the main manuscript, instead of fluid reasoning in the preregistration.

*Table S4.* Frequency of missing data in the ALSPAC cohort

| **Measures** | **Missing frequency** | **Percentage missing (%)** |
| --- | --- | --- |
| Peer Bullying | 9560 | 64.35 |
| Peer Relationship Problem | 8763 | 58.99 |
| Number of Close Friends | 8779 | 59.09 |
| School Issues ^a^ | 8877 | 59.75 |
| Lack of Family Support | 10991 | 73.98 |
| Sibling Bullying ^b^ | 7927 | 53.36 |
| IMD Income Score | 9012 | 60.66 |
| Working Memory | 11075 | 74.55 |
| Fluid Reasoning ^c^ | 9584 | 64.51 |
| Verbal Fluency | 9581 | 64.49 |

^a^ School issues is used here and in the main manuscript, instead of educational issues in the preregistration.

^b^ There is a high percentage of missing data for sibling bullying items since 476 participants reported that they do not have a sibling.

^c^ Fluid reasoning is used here and in the main manuscript, instead of fluid reasoning in the preregistration.

*Table S5.* Latent profile analysis fit statistics

| **Number of**  **profiles** | **BIC** | **SABIC** | **BLRT *p*-value** | **Entropy** |
| --- | --- | --- | --- | --- |
| *UKHLS* | | | | |
| 1 | 28138.41 | 28093.97 | NA | 1 |
| 2 | 27141.25 | 27071.43 | .010 | 1 |
| 3 | 27190.06 | 27094.84 | .812 | .59 |
| 4 | 26977.85 | 26857.23 | .010 | .91 |
| 5 | 27071.01 | 26925.01 | 1 | .70 |
| 6 | 27094.96 | 26923.57 | .010 | .68 |
| *ALSPAC* | | | | |
| 1 | 862799.84 | 862755.35 | NA | 1.00 |
| 2 | 860517.83 | 860447.91 | .010 | .43 |
| 3 | 859439.12 | 859343.78 | .010 | .68 |
| 4 | 858954.89 | 858834.13 | .010 | .59 |
| 5 | 858713.50 | 858567.32 | .010 | .60 |
| 6 | 858636.86 | 858465.26 | .010 | .49 |

*Table S6.* Profile prevalence of each LPA model in ALSPAC

| **Number of profiles** | **Number of participants (% prevalence)** | | | | | |
| --- | --- | --- | --- | --- | --- | --- |
|  | *1* | *2* | *3* | *4* | *5* | *6* |
| 1 | 14856  (100%) |  |  |  |  |  |
| 2 | 5366 (36.1%) | 9490 (63.9%) |  |  |  |  |
| 3 | 10866 (73.1%) | 3701 (24.9%) | 289 (1.9%) |  |  |  |
| 4 | 2542 (17.1%) | 2457 (16.5%) | 8592 (57.8%) | 1265 (8.5%) |  |  |
| 5 | 7933 (53.4%) | 2783 (18.7%) | 2517 (16.9%) | 632 (4.3%) | 991 (6.7%) |  |
| 6 | 719 (4.8%) | 2998 (20.2%) | 1525 (10.3%) | 2305 (15.5%) | 5251 (35.3%) | 2058 (13.9%) |

*Note: The number and percentage of participants in each profile per profile solution are shown. For example, the 1-profile solution contains 100% of the sample in 1 profile; the 2-profile solution contains 36.1% of the sample in profile 1 and 63.9% in profile 2.-profi*

*Table S7.* Comparison of social adversity scores between profiles in UKHLS.

| **Contrast** | **Estimate** | **SE** | **df** | ***t*-ratio** | ***p*-value** |
| --- | --- | --- | --- | --- | --- |
| *Peer Bullying* | | | | | |
| Low Adversity - Sibling Bullying | 0.00 | 1.53 | 486 | 0.00 | 1.000 |
| Low Adversity - Peer Difficulties | -37.21 | 1.39 | 486 | -26.70 | **<.0001** |
| Low Adversity - Poly-Adversity | -6.73 | 0.96 | 486 | -7.05 | **<.0001** |
| Sibling Bullying - Peer Difficulties | -37.21 | 1.95 | 486 | -19.12 | **<.0001** |
| Sibling Bullying - Poly-Adversity | -6.73 | 1.66 | 486 | -4.05 | **0.0001** |
| Peer Difficulties - Poly-Adversity | 30.48 | 1.54 | 486 | 19.80 | **<.0001** |
| *Peer Relationship Problems* | | | | | |
| Low Adversity - Sibling Bullying | 2.29 | 2.32 | 487 | 0.99 | 0.324 |
| Low Adversity - Peer Difficulties | -15.34 | 2.12 | 487 | -7.24 | **<.0001** |
| Low Adversity - Poly-Adversity | -6.39 | 1.45 | 487 | -4.40 | **<.0001** |
| Sibling Bullying - Peer Difficulties | -17.63 | 2.96 | 487 | -5.96 | **<.0001** |
| Sibling Bullying - Poly-Adversity | -8.68 | 2.52 | 487 | -3.44 | **0.0006** |
| Peer Difficulties - Poly-Adversity | 8.95 | 2.34 | 487 | 3.83 | **0.0001** |
| *Number of Close Friends* | | | | | |
| Low Adversity - Sibling Bullying | -1.93 | 3.43 | 461 | -0.56 | 0.574 |
| Low Adversity - Peer Difficulties | 3.68 | 3.15 | 461 | 1.17 | 0.243 |
| Low Adversity - Poly-Adversity | 0.67 | 2.15 | 461 | 0.31 | 0.755 |
| Sibling Bullying - Peer Difficulties | 5.61 | 4.39 | 461 | 1.28 | 0.202 |
| Sibling Bullying - Poly-Adversity | 2.60 | 3.74 | 461 | 0.70 | 0.487 |
| Peer Difficulties - Poly-Adversity | -3.01 | 3.48 | 461 | -0.86 | 0.388 |
| *Educational Issues* | | | | | |
| Low Adversity - Sibling Bullying | -5.94 | 3.01 | 489 | -1.97 | 0.049 |
| Low Adversity - Peer Difficulties | -4.65 | 2.75 | 489 | -1.69 | 0.092 |
| Low Adversity - Poly-Adversity | -14.37 | 1.88 | 489 | -7.63 | **<.0001** |
| Sibling Bullying - Peer Difficulties | 1.30 | 3.84 | 489 | 0.34 | 0.736 |
| Sibling Bullying - Poly-Adversity | -8.43 | 3.28 | 489 | -2.57 | 0.011 |
| Peer Difficulties - Poly-Adversity | -9.72 | 3.04 | 489 | -3.20 | 0.0015 |
| *Lack of Family Support* | | | | | |
| Low Adversity - Sibling Bullying | 0.00 | 0.59 | 487 | 0.00 | 1.000 |
| Low Adversity - Peer Difficulties | 0.00 | 0.54 | 487 | 0.00 | 1.000 |
| Low Adversity - Poly-Adversity | -34.90 | 0.37 | 487 | -94.65 | **<.0001** |
| Sibling Bullying - Peer Difficulties | 0.00 | 0.75 | 487 | 0.00 | 1.000 |
| Sibling Bullying - Poly-Adversity | -34.90 | 0.64 | 487 | -54.39 | **<.0001** |
| Peer Difficulties - Poly-Adversity | -34.90 | 0.59 | 487 | -58.68 | **<.0001** |

| *Sibling Bullying* | | | | | |
| --- | --- | --- | --- | --- | --- |
| Low Adversity - Sibling Bullying | -43.66 | 3.00 | 416 | -14.56 | **<.0001** |
| Low Adversity - Peer Difficulties | -10.78 | 2.88 | 416 | -3.74 | **0.0002** |
| Low Adversity - Poly-Adversity | -16.66 | 1.95 | 416 | -8.55 | **<.0001** |
| Sibling Bullying - Peer Difficulties | 32.88 | 3.92 | 416 | 8.39 | **<.0001** |
| Sibling Bullying - Poly-Adversity | 27.00 | 3.29 | 416 | 8.20 | **<.0001** |
| Peer Difficulties - Poly-Adversity | -5.88 | 3.19 | 416 | -1.85 | 0.066 |
| *Monthly Household Income* | | | | | |
| Low Adversity - Sibling Bullying | -1.00 | 1.07 | 486 | -0.94 | 0.350 |
| Low Adversity - Peer Difficulties | 0.57 | 0.98 | 486 | 0.58 | 0.560 |
| Low Adversity - Poly-Adversity | 1.36 | 0.67 | 486 | 2.01 | 0.045 |
| Sibling Bullying - Peer Difficulties | 1.58 | 1.37 | 486 | 1.15 | 0.250 |
| Sibling Bullying - Poly-Adversity | 2.36 | 1.17 | 486 | 2.02 | 0.044 |
| Peer Difficulties - Poly-Adversity | 0.79 | 1.08 | 486 | 0.72 | 0.469 |

*Note: P*-values in bold indicate significance at *alpha_Bonferroni_* = .001

*Table S8.* Comparison of social adversity scores between profiles in ALSPAC.

| **Contrast** | **Estimate** | **SE** | **df** | ***t*-ratio** | ***p*-value** |
| --- | --- | --- | --- | --- | --- |
| *Peer Bullying* | | | | | |
| Low Adversity - Sibling Bullying | -13.02 | 0.63 | 5292 | -20.79 | **<.0001** |
| Low Adversity - Peer Difficulties | -11.95 | 0.83 | 5292 | -14.39 | **<.0001** |
| Low Adversity - Poly-Adversity | -36.85 | 0.66 | 5292 | -56.15 | **<.0001** |
| Sibling Bullying - Peer Difficulties | 1.07 | 0.96 | 5292 | 1.12 | 0.264 |
| Sibling Bullying - Poly-Adversity | -23.83 | 0.81 | 5292 | -29.46 | **<.0001** |
| Peer Difficulties - Poly-Adversity | -24.90 | 0.98 | 5292 | -25.52 | **<.0001** |
| *Peer Relationship Problems* | | | | | |
| Low Adversity - Sibling Bullying | -3.88 | 0.31 | 6089 | -12.59 | **<.0001** |
| Low Adversity - Peer Difficulties | -32.00 | 0.40 | 6089 | -80.68 | **<.0001** |
| Low Adversity - Poly-Adversity | -2.66 | 0.33 | 6089 | -8.07 | **<.0001** |
| Sibling Bullying - Peer Difficulties | -28.12 | 0.46 | 6089 | -61.48 | **<.0001** |
| Sibling Bullying - Poly-Adversity | 1.22 | 0.40 | 6089 | 3.03 | 0.002 |
| Peer Difficulties - Poly-Adversity | 29.34 | 0.47 | 6089 | 62.12 | **<.0001** |
| *Number of Close Friends* | | | | | |
| Low Adversity - Sibling Bullying | 0.09 | 0.43 | 6073 | 0.22 | 0.829 |
| Low Adversity - Peer Difficulties | 6.44 | 0.56 | 6073 | 11.52 | **<.0001** |
| Low Adversity - Poly-Adversity | 3.28 | 0.47 | 6073 | 7.05 | **<.0001** |
| Sibling Bullying - Peer Difficulties | 6.35 | 0.64 | 6073 | 9.85 | **<.0001** |
| Sibling Bullying - Poly-Adversity | 3.19 | 0.57 | 6073 | 5.64 | **<.0001** |
| Peer Difficulties - Poly-Adversity | -3.17 | 0.67 | 6073 | -4.75 | **<.0001** |
| *Educational Issues* | | | | | |
| Low Adversity - Sibling Bullying | -6.25 | 0.35 | 5975 | -17.79 | **<.0001** |
| Low Adversity - Peer Difficulties | -8.22 | 0.47 | 5975 | -17.51 | **<.0001** |
| Low Adversity - Poly-Adversity | -11.24 | 0.36 | 5975 | -30.84 | **<.0001** |
| Sibling Bullying - Peer Difficulties | -1.97 | 0.54 | 5975 | -3.67 | **0.0002** |
| Sibling Bullying - Poly-Adversity | -4.99 | 0.45 | 5975 | -11.14 | **<.0001** |
| Peer Difficulties - Poly-Adversity | -3.02 | 0.55 | 5975 | -5.54 | **<.0001** |
| *Lack of Family Support* | | | | | |
| Low Adversity - Sibling Bullying | -5.89 | 0.44 | 3861 | -13.31 | **<.0001** |
| Low Adversity - Peer Difficulties | -1.33 | 0.60 | 3861 | -2.21 | 0.028 |
| Low Adversity - Poly-Adversity | -12.74 | 0.46 | 3861 | -27.58 | **<.0001** |
| Sibling Bullying - Peer Difficulties | 4.56 | 0.69 | 3861 | 6.62 | **<.0001** |
| Sibling Bullying - Poly-Adversity | -6.85 | 0.57 | 3861 | -12.02 | **<.0001** |
| Peer Difficulties - Poly-Adversity | -11.41 | 0.70 | 3861 | -16.26 | **<.0001** |
| *Sibling Bullying* | | | | | |
| Low Adversity - Sibling Bullying | -41.00 | 0.34 | 6925 | -121.02 | **<.0001** |
| Low Adversity - Peer Difficulties | -2.59 | 0.45 | 6925 | -5.72 | **<.0001** |
| Low Adversity - Poly-Adversity | -1.03 | 0.36 | 6925 | -2.87 | 0.004 |
| Sibling Bullying - Peer Difficulties | 38.41 | 0.52 | 6925 | 74.16 | **<.0001** |
| Sibling Bullying - Poly-Adversity | 39.97 | 0.44 | 6925 | 91.35 | **<.0001** |
| Peer Difficulties - Poly-Adversity | 1.56 | 0.53 | 6925 | 2.93 | 0.003 |
| *Monthly Household Income* | | | | | |
| Low Adversity - Sibling Bullying | 4.25 | 0.92 | 5840 | 4.61 | **<.0001** |
| Low Adversity - Peer Difficulties | 3.07 | 1.23 | 5840 | 2.49 | 0.013 |
| Low Adversity - Poly-Adversity | 8.34 | 0.96 | 5840 | 8.66 | **<.0001** |
| Sibling Bullying - Peer Difficulties | -1.18 | 1.41 | 5840 | -0.84 | 0.403 |
| Sibling Bullying - Poly-Adversity | 4.09 | 1.18 | 5840 | 3.47 | **0.0005** |
| Peer Difficulties - Poly-Adversity | 5.27 | 1.44 | 5840 | 3.67 | **0.0002** |

*Note: P*-values in bold indicate significance at *alpha_Bonferroni_* = .001

*Table S9.* Additional regression results for UKHLS and ALPSAC

| *UKHLS: Additional regression results, controlling for sex* | | | |
| --- | --- | --- | --- |
|  | **Working memory** | **Fluid reasoning** | **Verbal fluency** |
| Main effect sex | *F*(1, 411) = 0.10, *p* = .755 | *F*(1, 408) = 9.24, *p* = .003 | *F*(1, 416) = 2.29, *p* = .131 |
| R^2^ / R^2^ adjusted | 0.007 / -0.003 | 0.029 / 0.020 | 0.009 / -0.001 |
| *ALSPAC: Additional regression results, controlling for sex* | | | |
|  | **Working memory** | **Fluid reasoning** | **Verbal fluency** |
| Main effect sex | *F*(1, 3773) = 2.70, *p* = .101 | *F*(1,5258) = 8.83, *p* = .003 | *F*(1, 5261) = 9.05, *p* = .003 |
| R^2^ / R^2^ adjusted | 0.007 / 0.006 | 0.004 / 0.003 | 0.005 / 0.005 |
| *UKHLS: Additional regression results, not controlling for sex* | | | |
|  | **Working memory** | **Fluid reasoning** | **Verbal fluency** |
| R^2^ / R^2^ adjusted | 0.007 / -0.0005 | 0.007 / 0.0002 | 0.004 / -0.004 |
| *ALSPAC: Additional regression results, not controlling for sex* | | | |
|  | **Working memory** | **Fluid reasoning** | **Verbal fluency** |
| R^2^ / R^2^ adjusted | 0.007 / 0.006 | 0.002 / 0.001 | 0.004 / 0.003 |

*Table S10.* Estimated marginal means pairwise comparisons of cognitive functioning between social adversity profiles in ALSPAC, without controlling for sex.

| **Cognitive measure** |  | **Contrasts** | | | | | |
| --- | --- | --- | --- | --- | --- | --- | --- |
|  |  | *1 vs. 2* | *1 vs. 3* | *1 vs. 4* | *2 vs. 3* | *2 vs. 4* | *3 vs. 4* |
| Working Memory | est. | 2.90 | -1.57 | 3.56 | -4.47 | 0.67 | 5.14 |
|  | SE | 0.90 | 1.22 | 0.95 | 1.40 | 1.17 | 1.43 |
|  | df | 3777 | 3777 | 3777 | 3777 | 3777 | 3777 |
|  | *t*-ratio | 3.23 | -1.29 | 3.76 | -3.20 | 0.57 | 3.59 |
|  | *p* | **.001** | .197 | **<.001** | **.001** | .569 | **<.001** |
|  | *d* | 0.15 | -0.08 | 0.18 | -0.23 | 0.03 | 0.26 |
| Fluid Reasoning | est. | 0.21 | 0.69 | 1.56 | 0.49 | 1.35 | 0.86 |
|  | SE | 0.46 | 0.61 | 0.49 | 0.70 | 0.60 | 0.72 |
|  | df | 5268 | 5268 | 5268 | 5268 | 5268 | 5268 |
|  | *t*-ratio | 0.46 | 1.14 | 3.15 | 0.70 | 2.25 | 1.20 |
|  | *p* | .649 | .254 | **.002** | .486 | .024 | .230 |
|  | *d* | 0.01 | 0.04 | 0.08 | 0.02 | 0.07 | 0.04 |
| Verbal Fluency | est. | 1.60 | 1.08 | 2.01 | -0.52 | 0.41 | 0.93 |
|  | SE | 0.52 | 0.68 | 0.55 | 0.78 | 0.67 | 0.80 |
|  | df | 5271 | 5271 | 5271 | 5271 | 5271 | 5271 |
|  | *t*-ratio | 3.11 | 1.59 | 3.64 | -0.66 | 0.61 | 1.15 |
|  | *p* | **.002** | .112 | **<.001** | .507 | .540 | .249 |
|  | *d* | 0.08 | 0.05 | 0.10 | -0.03 | 0.02 | 0.05 |

*Note*. 1 = Low Adversity, 2 = Sibling Bullying, 3 = Peer Difficulties, 4 = Poly-Adversities. *P-*values in bold indicate significance after Bonferroni correction (adjusted alpha = .003).

*Table S11.* Descriptive statistics of adversity and cognition scores for each profile in UKHLS

| **Adversity** | **Low Adversity** | **Sibling Bullying** | **Peer Difficulties** | **Poly-Adversity** |
| --- | --- | --- | --- | --- |
| N (% prevalence) | 306 (62.1%) | 35 (7.1%) | 43 (8.7%) | 109 (22.1%) |
|  | Mean (SD) | | | |
| Peer Bullying | 33.33 (0.42) | 33.41 (0.00) | 70.56 (10.81) | 40.06 (16.84) |
| Peer Relationship Problems | 46.55 (12.51) | 43.64 (9.13) | 61.84 (13.78) | 52.80 (14.83) |
| Number of Close Friends | 25.48 (17.94) | 28.51 (16.30) | 20.23 (18.43) | 24.55 (21.45) |
| School Issues | 33.73 (15.50) | 41.59 (20.59) | 38.68 (14.81) | 48.43 (19.83) |
| Lack of Family Support | 33.26 (0.90) | 33.32 (0.00) | 33.33 (0.00) | 68.20 (7.01) |
| Sibling Bullying | 40.11 (13.30) | 75.98 (9.27) | 49.25 (22.59) | 56.65 (20.34) |
| Logged Household Income | 83.83 (6.10) | 85.05 (5.52) | 83.27 (3.91) | 82.41 (6.57) |
| Working Memory | 88.98 (20.36) | 89.29 (18.44) | 94.44 (16.29) | 88.28 (18.44) |
| Fluid Reasoning | 68.45 (15.98) | 67.26 (18.97) | 65.74 (20.29) | 64.96 (18.92) |
| Verbal Fluency | 52.84 (13.65) | 51.86 (18.61) | 53.68 (13.75) | 51.06 (11.73) |

*Table S12.* Descriptive statistics of adversity and cognition scores for each profile in ALSPAC

| **Adversity** | **Low Adversity** | **Sibling Bullying** | **Peer Difficulties** | **Poly-Adversity** |
| --- | --- | --- | --- | --- |
| N (% prevalence) | 8592 (57.8 %) | 2542 (17.1%) | 1265 (8.5%) | 2457 (16.5%) |
|  | Mean (SD) | | | |
| Peer Bullying | 32.04 (16.30) | 45.15 (20.21) | 45.36 (18.65) | 57.26 (17.06) |
| Peer Relationship Problems | 38.82 (10.12) | 42.95 (11.54) | 62.65 (8.70) | 42.17 (9.73) |
| Number of Close Friends | 22.86 (12.48) | 22.20 (12.87) | 15.84 (10.60) | 19.42 (11.76) |
| School Issues | 50.03 (9.53) | 56.34 (10.41) | 58.72 (9.73) | 59.18 (9.76) |
| Lack of Family Support | 31.71 (9.55) | 37.36 (11.08) | 33.54 (10.05) | 40.37 (11.77) |
| Sibling Bullying | 26.48 (12.86) | 61.21 (11.01) | 29.76 (13.88) | 29.85 (11.83) |
| IMD Income Score | 75.28 (25.58) | 71.85 (26.48) | 73.51 (25.81) | 69.29 (26.41) |
| Working Memory | 64.86(19.53) | 61.96(20.41) | 66.43(19.78) | 61.30(20.62) |
| Fluid Reasoning | 71.44(12.25) | 71.23(11.62) | 70.74(13.84) | 69.88(12.35) |
| Verbal Fluency | 64.85(13.72) | 63.24(13.17) | 63.76(14.59) | 62.83(14.32) |

*Table S13: Spearman’s correlation matrix of adversity variables in the UKHLS sample*

|  | 1 | 2 | 3 | 4 | 5 | 6 | 7 |
| --- | --- | --- | --- | --- | --- | --- | --- |
| 1. Peer bullying | - |  |  |  |  |  |  |
| 2. Number of friends | -.12* | - |  |  |  |  |  |
| 3. Lack of family support | .06 | -.06 | - |  |  |  |  |
| 4. Household income | -.06 | .14** | -.09* | - |  |  |  |
| 5. Sibling bullying | .05 | .03 | .23*** | .02 | - |  |  |
| 6. School issues | .16*** | -.03 | .30*** | -.05 | .21*** | - |  |
| 7. Peer relationship problems | .34*** | -.18*** | .14*** | -.15*** | .04 | .20*** | - |

*Note: * = p < .05, ** = p < .01, *** = p < .001*

*Table S14: Spearman’s correlation matrix of adversity variables in the ALSPAC sample*

|  | 1 | 2 | 3 | 4 | 5 | 6 | 7 |
| --- | --- | --- | --- | --- | --- | --- | --- |
| 1. Peer bullying | - |  |  |  |  |  |  |
| 2. Number of friends | -.09*** | - |  |  |  |  |  |
| 3. Lack of family support | .19*** | -.02 | - |  |  |  |  |
| 4. Household income | -.02 | .01 | -.03 | - |  |  |  |
| 5. Sibling bullying | .11*** | .01 | .16*** | .00 | - |  |  |
| 6. School issues | .19*** | -.10*** | .14*** | -.02 | .10*** | - |  |
| 7. Peer relationship problems | .07*** | -.11*** | .04 | -.01 | .03* | .16*** | - |

*Note: * = p < .05, ** = p < .01, *** = p < .001*
